# Complete Genome Sequences of Three Tomato Aspermy Virus Isolates in Japan

**DOI:** 10.1128/genomeA.00474-18

**Published:** 2018-05-31

**Authors:** Sota Inoue, Misato Tamura, Masashi Ugaki, Masashi Suzuki

**Affiliations:** a Laboratory of Bioresource Technology, Department of Integrated Biosciences, Graduate School of Frontier Sciences, The University of Tokyo, Kashiwa, Chiba, Japan

## Abstract

We report here the first complete nucleotide sequences of genomic RNAs of three Tomato aspermy virus (TAV) isolates in Japan. Analysis of these sequences showed that they have unique characteristics in RNAs 2 and 3. The Japanese isolates are similar to each other compared to other TAVs.

## GENOME ANNOUNCEMENT

Tomato aspermy virus (TAV) is a member of the genus Cucumovirus in the family Bromoviridae and has tripartite positive-sense single-stranded genomic RNAs (RNAs 1, 2, and 3). The TAV genome encodes five open reading frames. The 2b protein encoded by RNA 2 is a silencing suppressor (1). The 3a protein encoded by RNA 3 is essential for movement (2). TAV causes mosaic symptoms or necrosis in tomato plants and deformation in chrysanthemum flowers (3). TAV strains Go34 (TAV-Go34), G1 (TAV-G1), and H9 (TAV-H9) were isolated in Ibaraki, Gunma, and Shizuoka in Japan, respectively (4–6). We report here the complete nucleotide sequence of these TAVs.

The viruses were obtained from the National Agriculture and Food Research Organization (Japan) and propagated in Nicotiana benthamiana. The 5' and 3' terminal regions were determined by using a 5' RACE system for rapid amplification of cDNA ends version 2.0 (Invitrogen). Viral first-strand cDNA was synthesized using a Superscript III reverse transcriptase kit (Invitrogen), as described by Suzuki et al. (7). Synthesized cDNA was PCR amplified by TAV-specific primer pairs with restriction sites for cloning using KOD Plus version 2 DNA polymerase (Toyobo). The reverse transcription-PCR products were cloned into the pUC18 plasmid. The nucleotide sequences of at least six independent clones for each TAV isolate were determined and analyzed.

The identities of the nucleotide sequences between three Japanese isolates and the reported V-TAV (8–10), KC-TAV (11), and TAV-HN (12) isolates outside Japan were 98.4 to 99.6% for RNA 1, 95.1 to 99.3% for RNA 2, and 92.7 to 99.9% for RNA 3. V-TAV includes a repeat of 163 nucleotides at the 3' untranslated region of RNA 3 (13), but the Japanese isolates lacked these nucleotides. RNA 1 of three Japanese isolates includes a nucleotide insertion at the fifth nucleotide from the 5' end compared to that of V-TAV.

The length of the deduced 2 b protein was different among the strains. Those of TAV-Go34 and TAV-H9 comprise 104 amino acids, as is the case for three Iranian isolates (14) and TAV-HN (12), whereas that of TAV-G1 comprises 95 amino acids, as is the case for V-TAV (9) and KC-TAV (11). In the case of Cucumber mosaic virus, the length of the 2b protein coding region is different between its subgroups I and II. However, phylogenetic analysis of TAV RNAs 1, 2, and 3 using MEGA7 software with the neighbor-joining method showed that TAV-G1 is more similar to TAV-H9 than to V-TAV and KC-TAV and that TAV-H9 and TAV-Go34 are more similar to TAV-G1 than to TAV-HN and the Iranian isolates.

Interestingly, a single U nucleotide deletion at nucleotide position 94 was found in 58% of the TAV-Go34 RNA 3 clones, which resulted in frameshift and truncation in the encoded 3a protein. With a similar case in V-TAV, C-to-A substitution at nucleotide position 100 was found in 76% of the RNA 3 clones, which resulted in a stop codon and truncation in the 3a protein (15). The frequent mutation in the coding region of 3a and its truncation may be characteristic to TAV.

### Accession number(s).

The complete genome sequences of TAV strains G1, H9, and Go34 were deposited in DDBJ/GenBank under the accession numbers LC380669 to LC380677.
